# Seven new species of the genus *Trilacuna* Tong & Li, 2007 from Yunnan, China (Araneae, Oonopidae)

**DOI:** 10.3897/zookeys.821.29599

**Published:** 2019-01-31

**Authors:** Yanfeng Tong, Haifeng Chen, Shuchong Bai, Zhisheng Zhang, Shuqiang Li

**Affiliations:** 1 College of Life Science, Shenyang Normal University, Shenyang 110034, China Shenyang Normal University Shenyang China; 2 College of Life Science, Langfang Normal University, Langfang 065000, Hebei Province, China Langfang Normal University Langfang China; 3 Key Laboratory of Eco-environments in Three Gorges Reservoir Region (Ministry of Education), School of Life Sciences, Southwest University, Chongqing 400715, China Southwest University Chongqing China; 4 Institute of Zoology, Chinese Academy of Sciences, Beijing 100101, China Institute of Zoology, Chinese Academy of sciences Beijing China

**Keywords:** Asia, Oonopinae, southwestern China, spider, taxonomy

## Abstract

Seven new species of the genus *Trilacuna* Tong & Li, 2007: *Trilacunabawan* Tong, Zhang & Li, **sp. n.** (male, female), *T.datang* Tong, Zhang & Li, **sp. n.** (male, female), *T.fugong* Tong, Zhang & Li, **sp. n.** (male, female), *T.gongshan* Tong, Zhang & Li, **sp. n.** (male, female), *T.longling* Tong, Zhang & Li, **sp. n.** (male, female), *T.wuhe* Tong, Zhang & Li, **sp. n.** (male, female), and *T.xinping* Tong, Zhang & Li, **sp. n.** (male, female) are described from Yunnan Province, China.

## Introduction

Oonopidae is a diverse spider family with 1807 extant described species in 115 genera (WSC 2019; Li and Quan 2017). They have a nearly worldwide distribution, occurring mainly in the leaf litter, under bark, and in the tree canopy (Jocqué and Dippenaar-Schoeman 2006; Ubick and Dupérré 2017).

*Trilacuna* Tong & Li, 2007 currently comprises 22 species. Members of this genus are known from Iran, China, and south to Sumatra (WSC 2019). Only four species of *Trilacuna* are known in China: *T.angularis* Tong & Li, 2007, *T.simianshan* Tong & Li, 2018 and *T.songyuae* Tong & Li, 2018 from Chongqing, and *T.rastrum* Tong & Li, 2007 from Yunnan Province. In this paper seven new *Trilacuna* species collected form Yunnan Province, are described and illustrated.

## Materials and methods

The specimens were examined using a Leica M205C stereomicroscope. Details were studied under an Olympus BX51 compound microscope. Photos were made with a Canon EOS 550D zoom digital camera (18 megapixels) mounted on an Olympus BX51 compound microscope. Vulvae were cleared in lactic acid. Scanning electron microscope images (SEM) were taken under high vacuum with a Hitachi S-4800 after critical point drying and gold-palladium coating. All measurements were taken using an Olympus BX51 compound microscope and are in millimeters.

The following abbreviations are used in the text and figures:

ap apodeme;

as anterior sclerite (T-shaped sclerite);

bep basal ear-shaped projection;

bll blade-like lobes;

blp basal leaf-shaped projection;

bmb broad medial branch;

boc booklung covers;

bth basal thin “hairs”;

clh cluster of long hairs;

csl comb-shaped lobes;

css cluster of short setae;

dbe distal bending;

dbh dorsal brush of “hairs”;

dbl distal broad lobe;

ddo dark dot;

dha distal “hairs”;

dmp distal medial plate;

dpr distal projection;

dsh distal short “hairs”;

dth distal thick “hairs”;

ehb elevated hair base;

fll finger-like lobes;

glo globular structure;

grl rows of lobes in basal ventral groove;

gro grooves;

hsc horseshoe-shaped sclerite;

lbh long, brush of “hairs”;

lcb lateral curved branch;

ldi labium deep incision;

ldp large distal plate;

lh lateral “hairs”;

lha long hairs;

lmb long medial branch;

lth long thick “hairs”;

lts long, very thick setae;

mel median elevation;

psp posterior spiracle;

rls rows of long setae;

sar sclerotized, recurved arches;

sdb slightly curved distal branch;

sis short, italic thick setae;

slh small hole;

sri small ridges;

ssc stick-like sclerite;

tba transverse bars;

tll tooth-like lobes;

tsc transverse sclerite;

vbl ventral broad lobes;

wri wrinkles.

Type material is deposited in Shenyang Normal University (**SYNU**) in Shenyang, China.

## Taxonomy

### Family Oonopidae Simon, 1890

#### Genus Trilacuna

Taxon classificationAnimaliaAraneaeOonopidae

Tong & Li, 2007

Trilacuna Tong & Li, 2007: 333; Grismado et al. 2014: 26.

##### Type species.

*Trilacunarastrum* Tong & Li, 2007.

##### Comments.

This genus was originally diagnosed by the enlarged male palpal femora, the very complicated embolus-conductor complex, the branched endites in males, and the notched labium (Tong and Li 2007). These characters were later recognized as shared by a more inclusive group, the “*Dysderoides* complex”, including *Bannana* Tong & Li, 2015, *Dysderoides* Fage, 1946, *Himalayana* Grismado, 2014, and *Trilacuna* (Grismado et al. 2014; Tong and Li 2015). The genus *Bannana* is distributed only in Xishuangbanna, Yunnan Province, China; *Dysderoides* species were known from caves from northern India and Thailand; species of *Himalayana* were known only in Nepal and northern India; while those of *Trilacuna* have a wider distribution from Iran to the Korean Peninsula.

**Figure 1.**
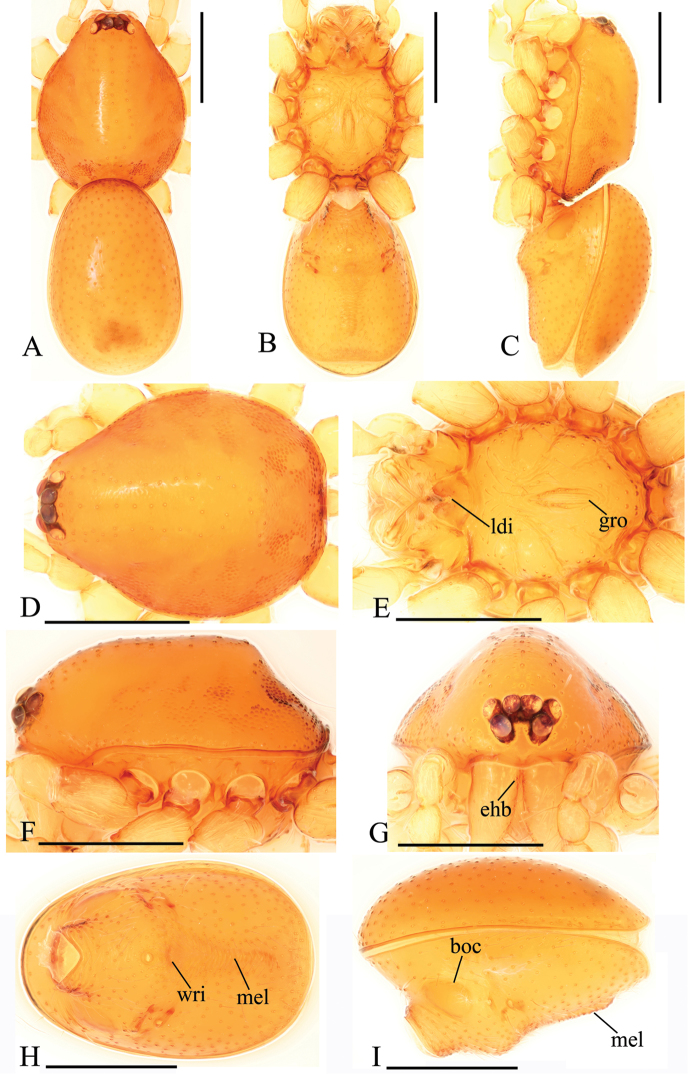
*Trilacunabawan* sp. n., male. **A–C** habitus, dorsal, ventral and lateral views **D–G** prosoma, dorsal, ventral, lateral and anterior views **H, I** abdomen, ventral and lateral views. Abbreviations: boc = booklung covers; ehb = elevated hair base; gro = grooves; ldi = labium deep incision; mel = median elevation; wri = wrinkles. Scale bars: 0.4 mm.

The genus *Trilacuna* can be distinguished from the other genera of the *Dysderoides* complex by the females having a long postepigastric scutum, covering almost the whole ventral abdomen (Fig. 3G), and males usually lacking the furrow connecting the posterior tracheal spiracles (Fig. 1H). The females of the other genera of the *Dysderoides* complex, *Bannana*, *Dysderoides*, and *Himalayana* have a very short postepigastric scutum, only around the epigastric furrow (Grismado et al. 2014: fig. 12E, 69H; Tong and Li 2015: fig. 5G), and males have the furrow connecting the posterior tracheal spiracles (Grismado et al. 2014: fig. 59D; Tong and Li 2015: fig. 1G).

##### Composition.

29 species, 7 of them are described here.

##### Distribution.

Iran to Korean Peninsula.

#### Trilacuna bawan

Taxon classificationAnimaliaAraneaeOonopidae

Tong, Zhang & Li sp. n.

http://zoobank.org/C097B8B5-259B-4E96-AE13-94B2CB9A0E9E

**Figure 2.**
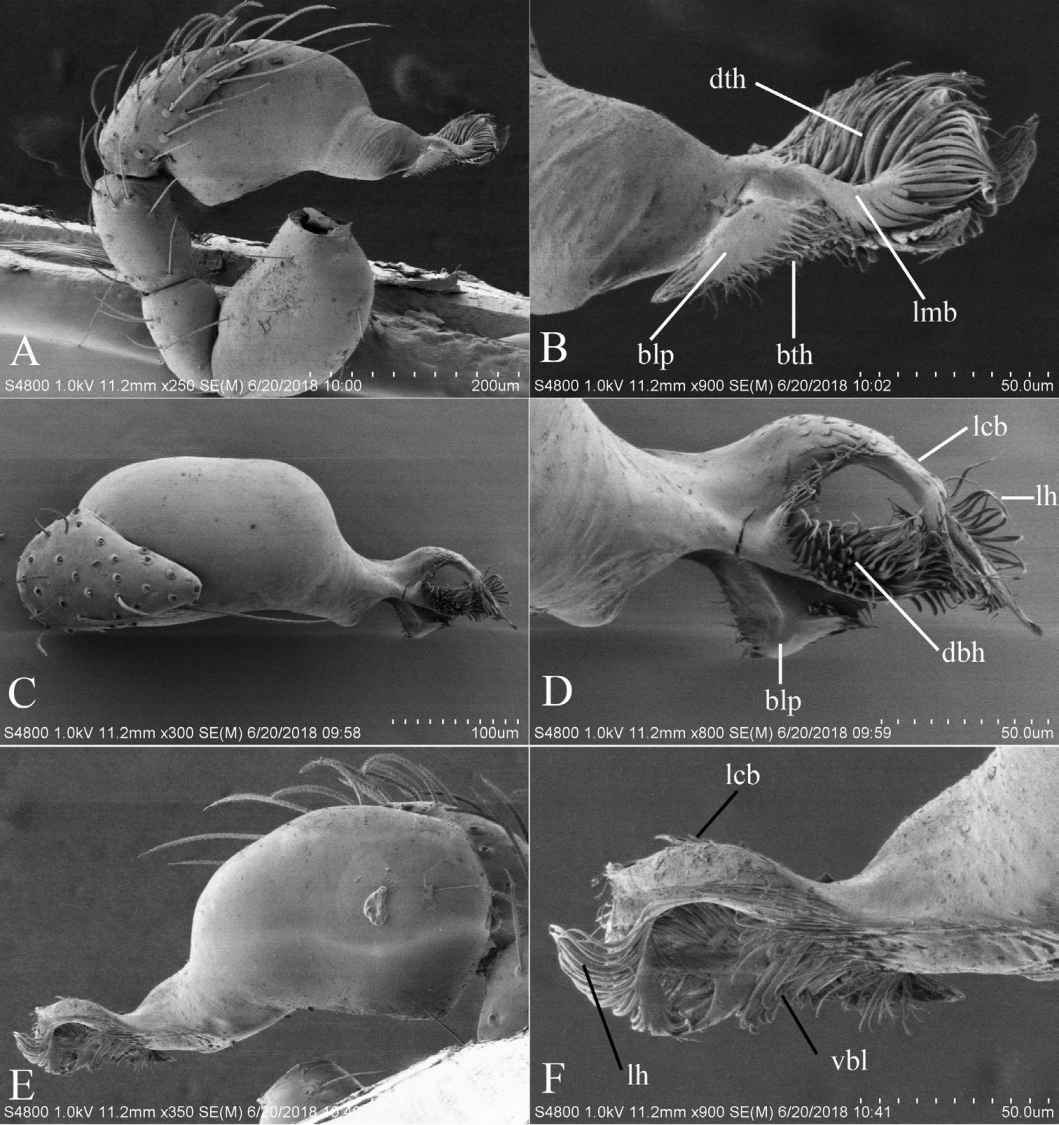
*Trilacunabawan* sp. n., left male palp. **A** prolateral view **B, D, F** distal part of palpal bulb, prolateral, dorsal and retrolateral views **C, E** palpal bulb, dorsal and retrolateral views. Abbreviations: blp = basal leaf-shaped projection; bth = basal thin “hairs”; dbh = dorsal brush of “hairs”; dth =distal thick “hairs”; lcb = lateral curved branch; lh = lateral “hairs”; lmb = long medial branch; vbl = ventral broad lobes.

**Figure 3.**
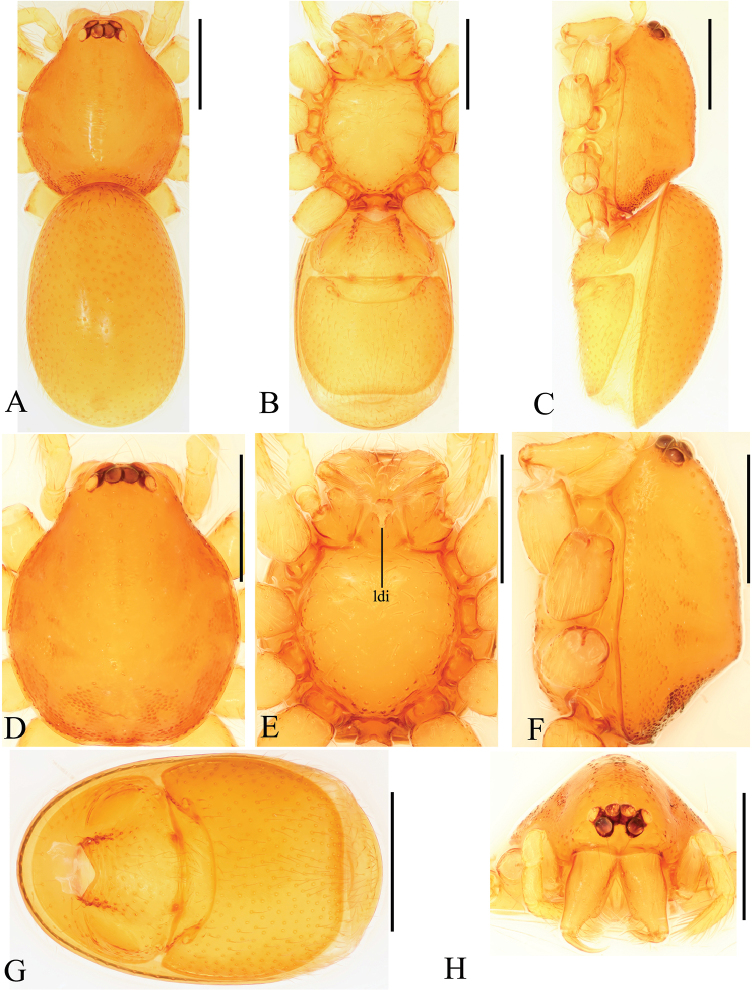
*Trilacunabawan* sp. n., female. **A–C** habitus, doral, ventral and lateral views **D–F, H** prosoma, dorsal, ventral, lateral and anterior views **G** abdomen, ventral view. Abbreviation: ldi = labium deep incision. Scale bars: 0.4 mm.

Figs 1 , 2 , 3 , 22A, B , 24A, B , 26A

##### Type material.

**Holotype** ♂ (SYNU-254), China, Yunnan Province, Baoshan City, Bawan Town, Dasheyao, 13.II.2011, Zongxu Li & Luyu Wang. **Paratypes**: 3♀ (SYNU-252), same data as holotype; 1♂ (SYNU-253), same locality as holotype, 14.II.2011.

##### Etymology.

The specific name is a noun in apposition taken from the type locality.

##### Diagnosis.

Males of the new species is similar to *T.hansanensis* Seo, 2017, but can be distinguished by the following characters: sternum with grooves (gro) on posterior area (Fig. 1E); epigastric region strongly elevated, then followed by a narrow, slightly median elevation (mel) (Fig. 1I); embolus system with a leaf-shaped projection on basal part (blp) (Fig. 2B, D). *T.hansanensis* has a pair of chitinized ridges on posterior area of sternum (Seo 2017: fig. 1B), epigastric region and ventral surface of abdomen not elevated (Seo 2017: fig. 1B), and has a strongly bent terminal hook on basal part of bulb (Seo 2017: fig. 1K). Females of the new species are similar to those of *T.songyuae* Tong & Li, 2018, but can be distinguished by the absence of the large dark knob marking in ventral surface of genital area (Tong et al. 2018: fig. 10H).

##### Description.

**Male**. Body yellow-brown, chelicerae and sternum lighter, legs yellow. Habitus as in Fig. 1A–C. Body length 1.76; carapace 0.88 long, 0.74 wide; abdomen 0.97 long, 0.64 wide. Carapace sides granulate; lateral margin rebordered (Fig. 1D). Six eyes, well developed, nearly equal sized, arranged in a compact group; ALE–PLE separated by less than ALE radius, PME touching each other; posterior row recurved from above, procurved from front (Fig. 1D, G). ALE separated from edge of carapace by 1.1 diameters. Mouthparts (Figs 1E, G, 26A): chelicerae straight, proximal region with one hair with elevated hair base; labium rectangular, anterior margin deeply incised (ldi); endites slender, distally only slightly branched (sdb). Sternum surface smooth, with several grooves (gro) on posterior area (Fig. 1E). Abdomen as in Fig. 1I. Leg spination (all spines longer than segment width): legs I-II: tibia: v2-2-2-2-0, metatarsus: v2-2-0. **Genitalia**. Sperm pore situated at level of anterior spiracles; with several wrinkles (wri) between the posterior spiracles (Fig. 1H); epigastric region strongly elevated in lateral view, then followed by a narrow, slightly median elevation (mel) (Fig. 1I). Palp (Figs 2, 22A, B): orange. 0.46 long (0.19, 0.11, 0.12, 0.17). Femur 0.19 long, 0.13 wide (width/length = 0.68) (Fig. 22A, B). Tip of cymbium with long setae, almost as long as cymbium. Bulb oval, stout, tapering apically. Embolus system (Fig. 2B, D, F) with a leaf-shaped projection on base (blp) prolaterally; with a long medial branch (lmb) and a lateral curved branch (lcb) dorsally; all these structures surrounded by numerous hair-like structures.

**Female**. As in male except as noted. Habitus as in Fig. 3A–C. Slightly larger than male. Body length 1.89; carapace 0.85 long, 0.75 wide; abdomen 1.12 long, 0.73 wide. Endites unmodified; sternum without grooves on middle area; ventral side of abdomen unmodified. **Genitalia**. Ventral view (Figs 3G, 24A): with recurved, strongly sclerotized arches (sar) anterior to the spiracles. Dorsal view (Fig. 24B): with narrow, transverse sclerite (tsc); with an anterior T-shaped sclerite (as) and a posterior small globular structure (glo). Transverse bars (tba) nearly straight, with two short, lateral apodemes (ap).

##### Distribution.

Known only from the type locality.

#### Trilacuna datang

Taxon classificationAnimaliaAraneaeOonopidae

Tong, Zhang & Li sp. n.

http://zoobank.org/E680C826-455B-4E90-912C-5D43CD8D4A26

**Figure 4.**
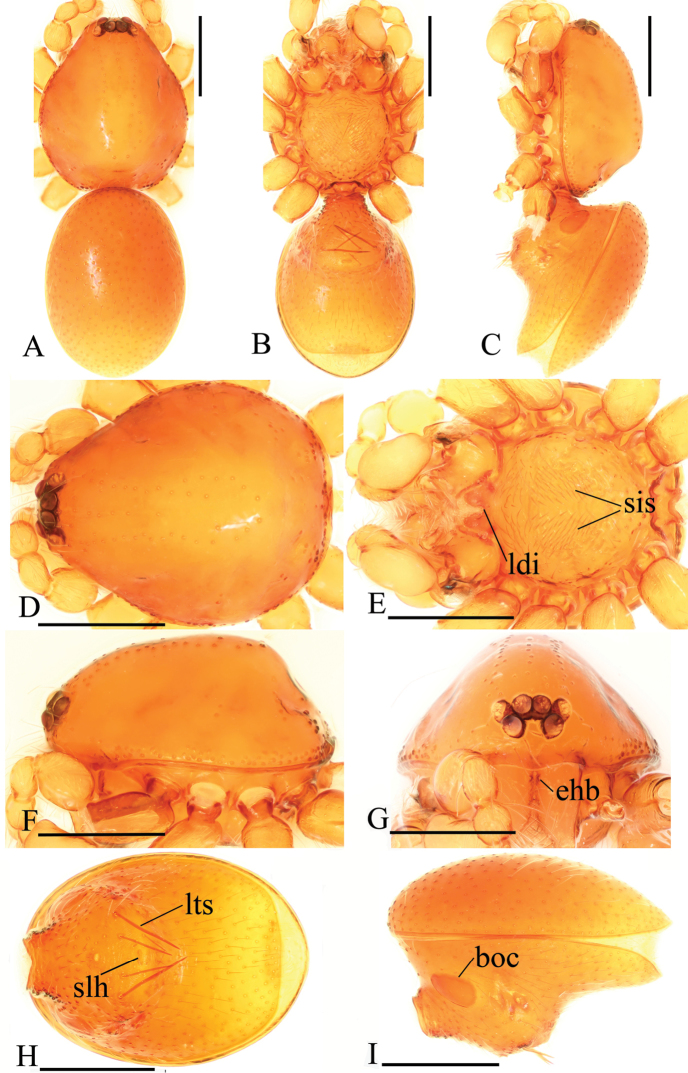
*Trilacunadatang* sp. n., male. **A–C** habitus, dorsal, ventral and lateral views **D–G** prosoma, dorsal, ventral, lateral and anterior views **H, I** abdomen, ventral and lateral views. Abbreviations: boc = booklung covers; ehb = elevated hair base; ldi = labium deep incision; lts = long, very thick setae; sis = short, italic thick setae; slh = small hole. Scale bars: 0.4 mm.

**Figure 5.**
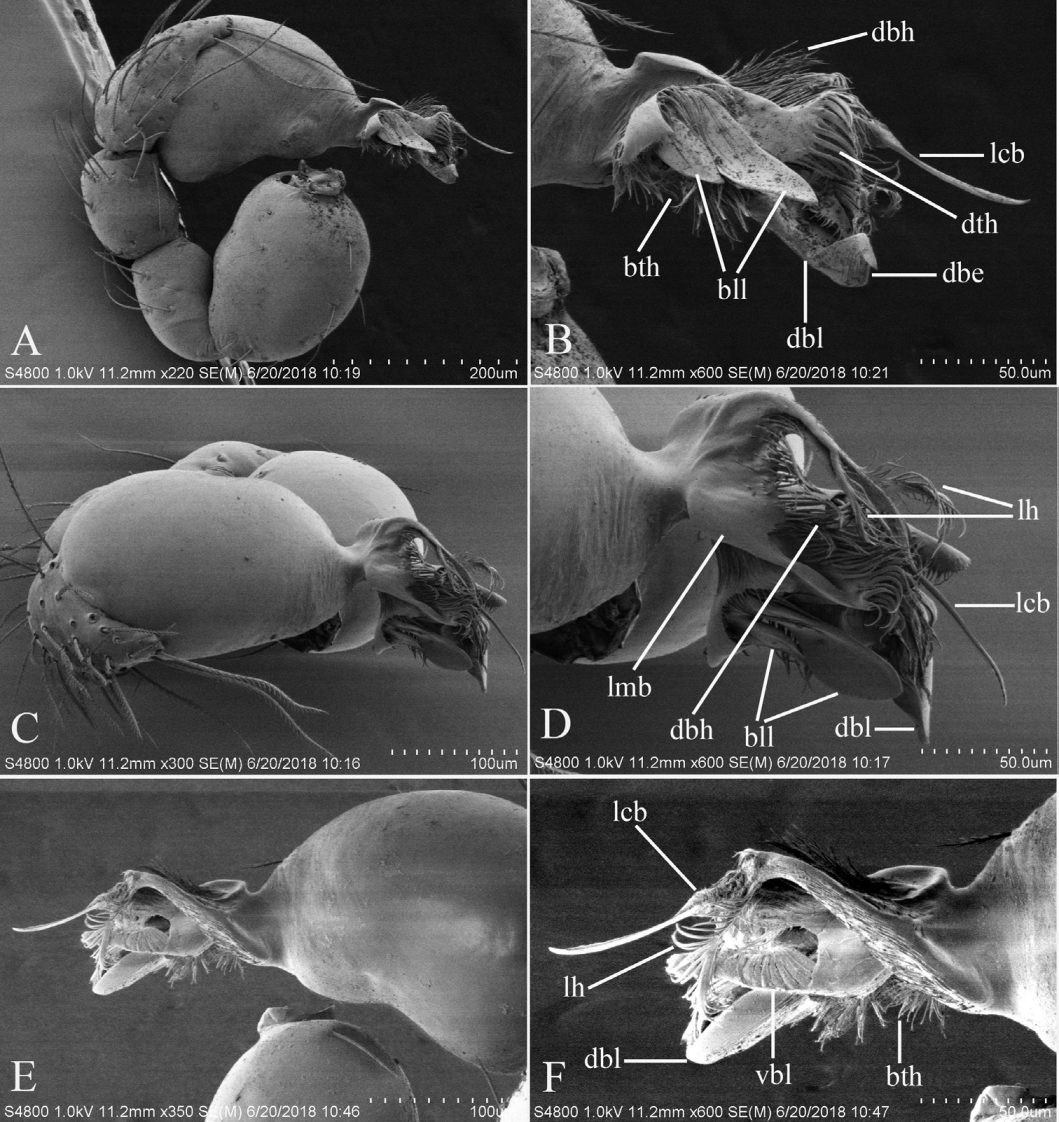
*Trilacunadatang* sp. n., left male palp. **A** prolateral view **B, D, F** distal part of palpal bulb, prolateral, dorsal and retrolateral views **C, E** palpal bulb, dorsal and retrolateral views. Abbreviations: bll = blade-like lobes; bth = basal thin “hairs”; dbe = distal bending; dbh = dorsal brush of “hairs”; dbl = distal broad lobe; dth = distal thick “hairs”; lcb = lateral curved branch; lh = lateral “hairs”; lmb = long medial branch; vbl = ventral broad lobes.

**Figure 6.**
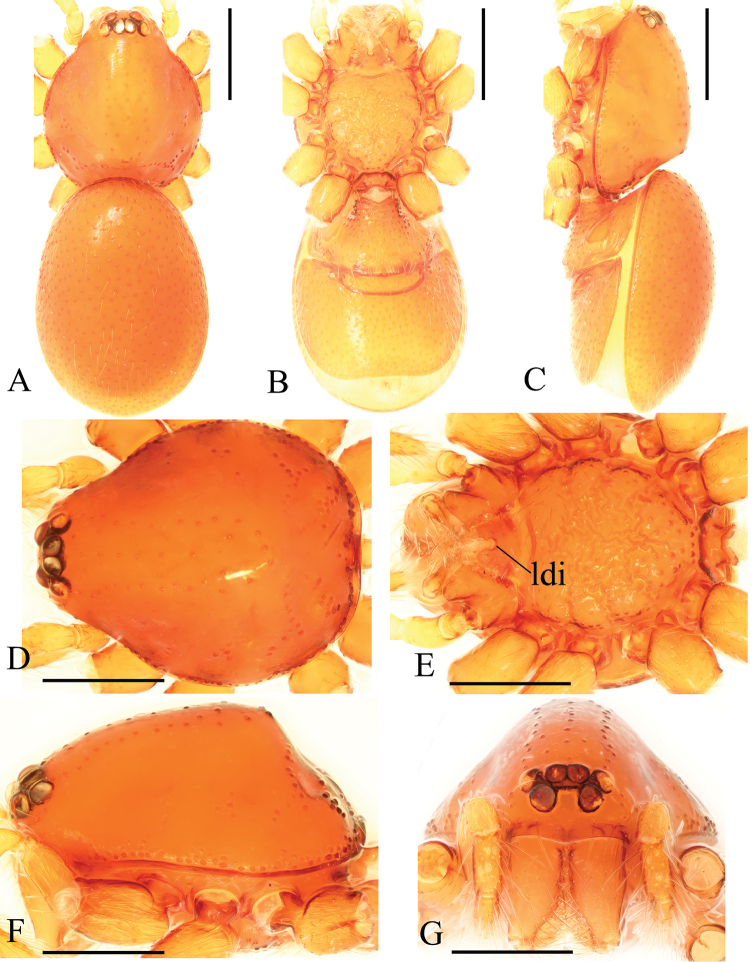
*Trilacunadatang* sp. n., female. **A–C** habitus, dorsal, ventral and lateral views **D–G** prosoma, dorsal, ventral, lateral and anterior views. Abbreviation: ldi = labium deep incision. Scale bars: 0.4 mm.

Figs 4 , 5 , 6 , 22C, F , 24C, D , 26B

##### Type material.

Holotype ♂ (SYNU-240), China, Yunnan Province, Baoshan City, Tengchong County, Jietou Town, Datang Village, Dahelingganjiao, 23.II.2011, Zongxu Li & Luyu Wang. **Paratypes**: 6♀, 1♂ (SYNU-241), same data as holotype; 1♂, 3♀ (SYNU-242), same locality as holotype, 21.II.2011; 1♂ (SYNU-243), same locality as holotype, 24.II.2011.

##### Etymology.

The specific name is a noun in apposition taken from the type locality.

##### Diagnosis.

The new species is similar to *T.rastrum* Tong & Li, 2007, but males can be distinguished by the strongly elevated epigastric region (Fig. 4I), the long, very thick setae (lts) between the anterior spiracles (Fig. 4H) and 2 basal broad blade-like lobes (bll) and one long distal broad lobe (dbl) of embolus system (Fig. 5B), and the females by the smooth carapace and rugose surface of the sternum. *Trilacunarastrum* males have flat epigastric region, without thick setae between the anterior spiracles, and have rake-like lobes of embolus system, and females have granulated sides of carapace and pitted surface of the sternum.

##### Description.

**Male**. Body yellow-brown, chelicerae and sternum lighter, legs yellow. Habitus as in Fig. 4A–C. Body length 1.87; carapace 0.89 long, 0.76 wide; abdomen 0.99 long, 0.72 wide. Carapace sides smooth, lateral margin rebordered (Fig. 4D). Eyes similar to those of *T.bawan* sp. n. (Fig. 4D, G). ALE separated from edge of carapace by 1.3 diameters. Mouthparts (Figs 4E, G, 26B) similar to those of *T.bawan* sp. n. Sternum surface smooth, with many short, italic thick setae (sis) on middle area (Fig. 4E). Abdomen as in Fig. 4I. Leg spination (all spines longer than segment width): legs I-II: tibia: v2-2-2-2-0, metatarsus: v2-2-0. **Genitalia**. Sperm pore situated in front of anterior spiracles; with four long, very thick setae (lts) between the anterior spiracles; with a small hole (slh) between the posterior spiracles (Fig. 4H); epigastric region strongly elevated (Fig. 4I) in lateral view. Palp (Figs 5, 22C, F): orange. 0.46 long (0.15, 0.08, 0.11, 0.12). Femur 0.15 long, 0.09 wide (width/length = 0.6) (Fig. 22C, F). Tip of cymbium with long setae, longer than cymbium. Bulb oval, stout, tapering apically. Embolus system (Fig. 5B, D, F) with 2 basal broad blade-like lobes (bll) and one long distal broad lobe (dbl) prolaterally, the tip of the distal lobe sharply bending (dbe); with a lateral curved branch (lcb) and a long medial branch (lmb) dorsally; with numerous brush of “hairs” (dbh) on dorsal branch and lateral “hairs” (lh) on small branches derived from the lateral curved branch (lcb).

**Female**. As in male except as noted. Habitus as in Fig. 6A–C. Body length 1.87; carapace 0.85 long, 0.74 wide; abdomen 1.09 long, 0.81 wide. Endites unmodified; sternum surface strongly rugose; ventral side of abdomen unmodified. **Genitalia**. Ventral view (Fig. 24C): with recurved, strongly sclerotized arches (sar) anterior to the spiracles; grooves connected posterior spiracles heavily sclerotized. Dorsal view (Fig. 24D): with narrow, transversally elongated sclerite (tsc); with an anterior T-shaped sclerite (as) and a posterior small globular structure (glo). Transverse bars (tba) slightly arched, with two short, lateral apodemes (ap).

##### Distribution.

Known only from the type locality.

#### Trilacuna fugong

Taxon classificationAnimaliaAraneaeOonopidae

Tong, Zhang & Li sp. n.

http://zoobank.org/918ACACD-6775-43C9-BF67-9139BFFDF847

**Figure 7.**
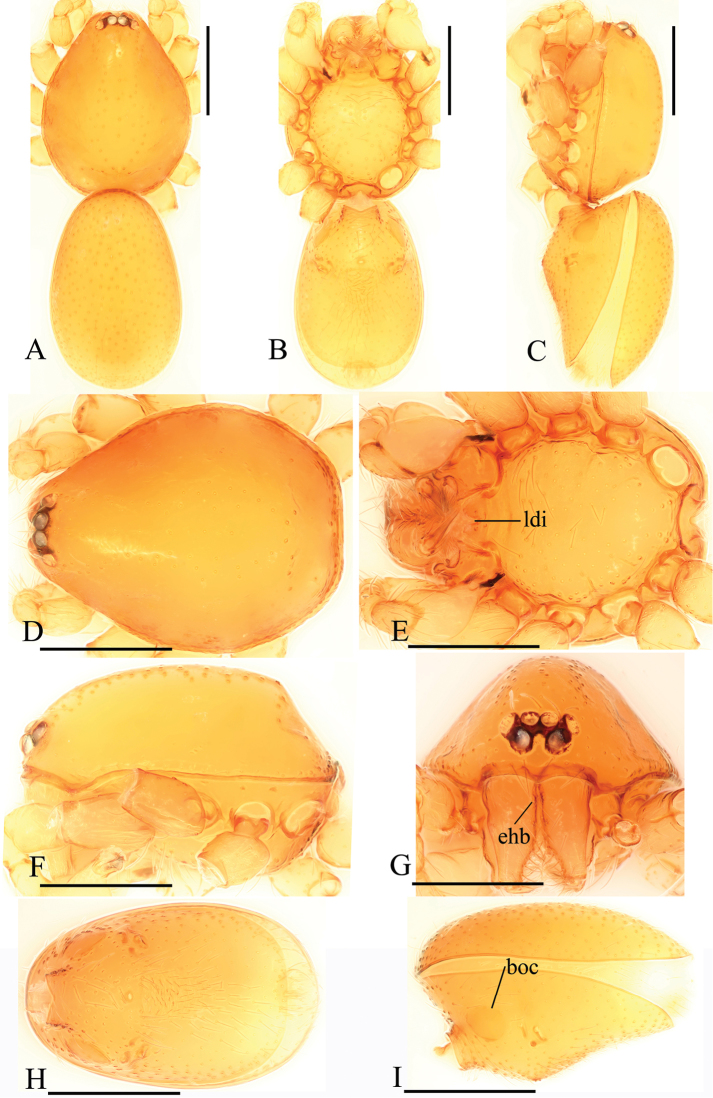
*Trilacunafugong* sp. n., male. **A–C** habitus, dorsal, ventral and lateral views **D–G** prosoma, dorsal, ventral, lateral and anterior views **H, I** abdomen, ventral and lateral views. Abbreviations: boc = booklung covers; ehb = elevated hair base; ldi = labium deep incision. Scale bars: 0.4 mm.

**Figure 8.**
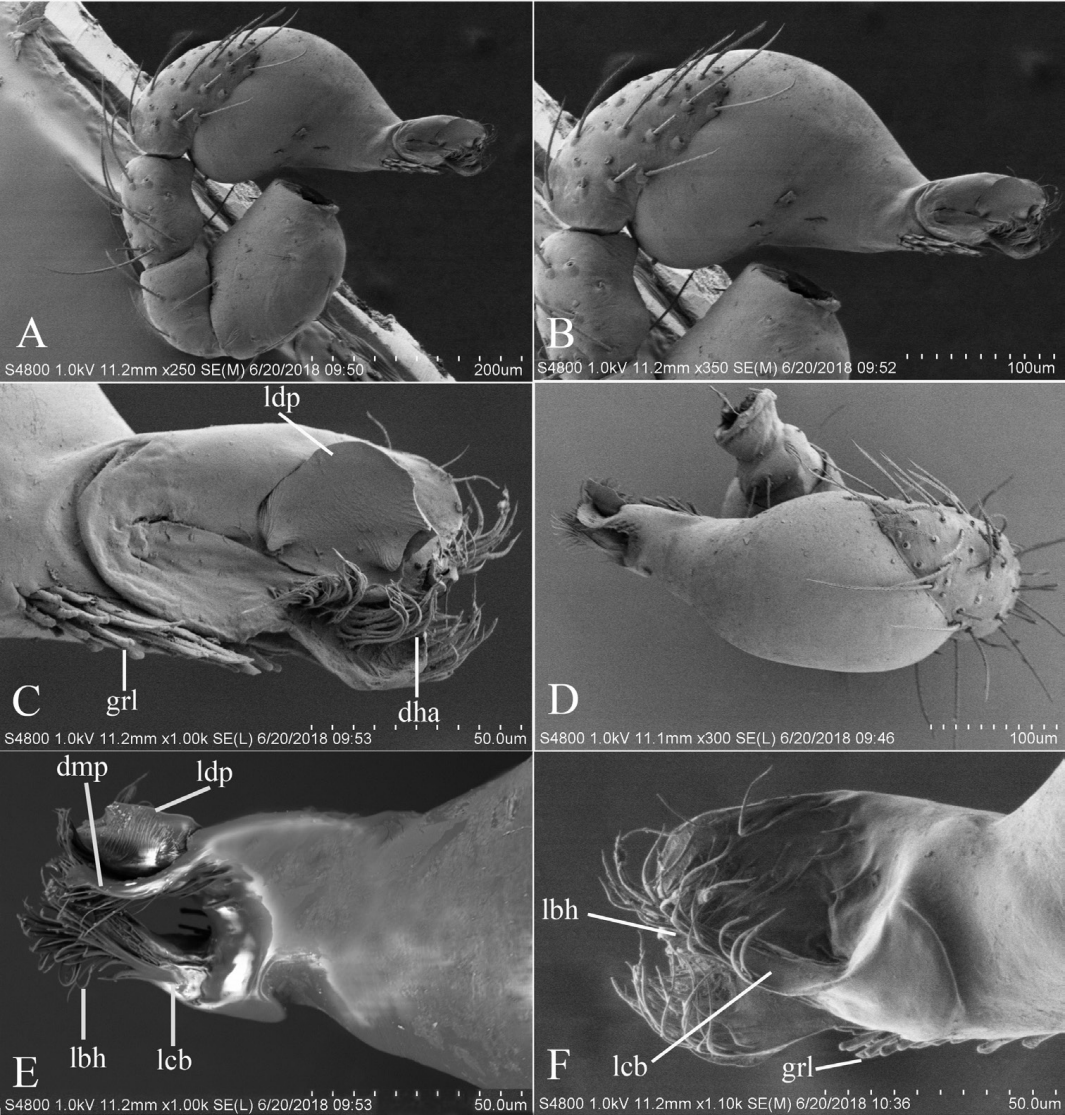
*Trilacunafugong* sp. n., left male palp. **A** prolateral view **B, D** palpal bulb, prolateral and dorsal views **C, E, F** distal part of palpal bulb, prolateral, dorsal and retrolateral views. Abbreviations: dha = distal “hairs”; dmp = distal medial plate; grl = rows of lobes in basal ventral groove; lbh = long, brush of “hairs”; lcb = lateral curved branch; ldp = large distal plate.

**Figure 9.**
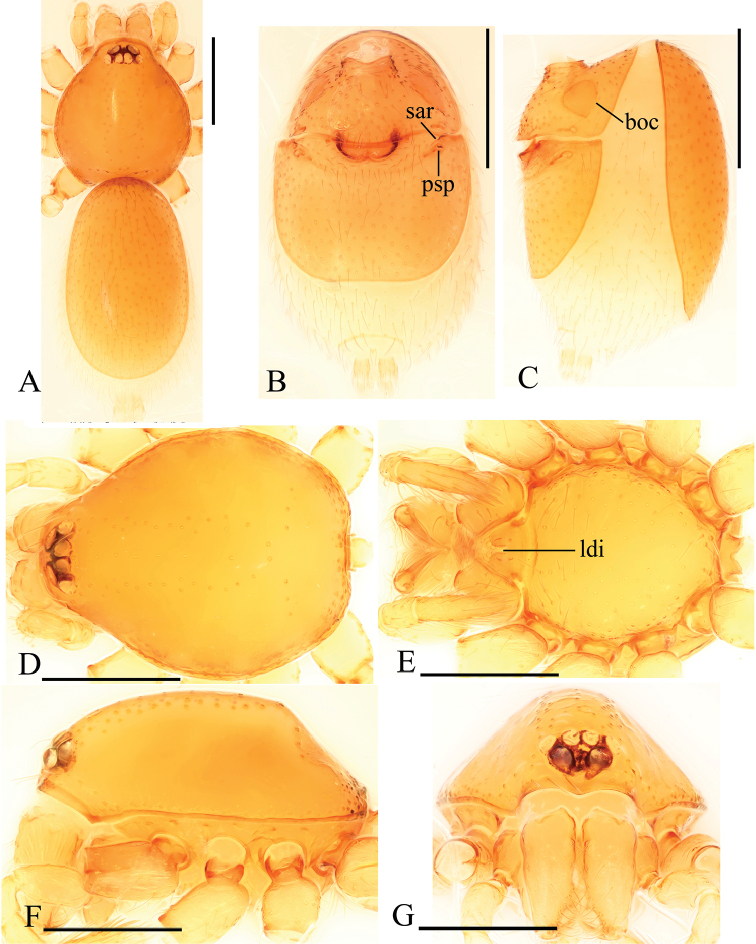
*Trilacunafugong* sp. n., female. **A** habitus, dorsal view **B, C** abdomen, ventral and lateral views **D–G** prosoma, dorsal, ventral, lateral and anterior views. Abbreviations: boc = booklung covers; ldi = labium deep incision; psp = posterior spiracle; sar = sclerotized, recurved arches. Scale bars: 0.4 mm.

Figs 7 , 8 , 9 , 22D, E , 24E, F , 26C

##### Type material.

**Holotype** ♂ (SYNU-250), China, Yunnan Province, Nujiang Lisu Autonomous Prefecture, Fugong County, Pihe Town, 11.III.2011, Zongxu Li & Luyu Wang. **Paratypes**: 1♀, 2♂ (SYNU-251), same data as holotype.

##### Etymology.

The specific name is a noun in apposition taken from the type locality.

##### Diagnosis.

Males of the new species is similar to *T.werni* Eichenberger, 2011, but can be distinguished by the several rows of lobes on ventral groove (grl) of the bulb (Fig. 8C), the large distal plate (ldp) of embolus system (Fig. 8C), and the smooth carapace and sternum (Fig. 7D, E) *vs.* only one row of lobes in a venrtal groove without large distal plate on embolus system in *T werni* (Eichenberger and Kranz-Baltensperger 2011: fig. 12E‒F, I), and granulated carapace and reticulated sternum in *T.werni* (Eichenberger and Kranz-Baltensperger 2011: fig. 10D–G). Females of the new species can be distinguished from all other *Trilacuna* species by the horseshoe-shaped sclerite (hsc) of the endogyne (Fig. 24F), the females of all the known species have transversally elongated sclerite of the endogyne (e.g., Figs 24B, D, H, J, 25B, D).

##### Description.

**Male**. Body yellow, chelicerae, sternum and legs lighter. Habitus as in Fig. 7A–C. Body length 1.59; carapace 0.78 long, 0.64 wide; abdomen 0.86 long, 0.55 wide. Carapace sides smooth, lateral margin rebordered (Fig. 7D). Eyes: ALE largest, PME smallest (Fig. 7D, G). ALE separated from edge of carapace by approximately one diameter. Mouthparts as in Figs 7E, G, 26C, endites with a small distal projection (dpr). Sternum surface smooth (Fig. 7E). Abdomen as in Fig. 7I. Leg spination (all spines longer than segment width): legs I-II: tibia: v2-2-2-0, metatarsus: v2-2-0. **Genitalia**. Epigastric region sharply elevated from lateral view (Fig. 7I), sperm pore situated at level of anterior spiracles. Palp (Figs 8, 22D, E): orange. 0.46 long (0.15, 0.08, 0.11, 0.12). Femur 0.15 long, 0.09 wide (width/length = 0.6) (Fig. 22D, E). Cymbium without long setae. Bulb oval, stout, tapering apically. Embolus system (Fig. 8C, E, F) with rows of lobes in basal ventral groove (grl) and a large distal plate (ldp); with a distal medial plate (dmp) and a short lateral curved branch (lcb), surrounded by long, brush of “hairs” (lbh).

**Female**. As in male except as noted. Habitus as in Fig. 9A. Slightly larger than male. Body length 1.68; carapace 0.71 long, 0.63 wide; abdomen 1.01 long, 0.63 wide. Endites unmodified; epigastric region not sharply elevated from lateral view. **Genitalia**. Ventral view (Figs 9B, 24E): with recurved, strongly sclerotized arches (sar) anterior to the spiracles. Dorsal view (Fig. 24F): with an anterior stick-like sclerite (ssc) and a posterior horseshoe-shaped sclerite (hsc).

##### Distribution.

Known only from the type locality.

#### Trilacuna gongshan

Taxon classificationAnimaliaAraneaeOonopidae

Tong, Zhang & Li sp. n.

http://zoobank.org/DD6BC9A6-B90B-4789-84AD-BCE229277B42

**Figure 10.**
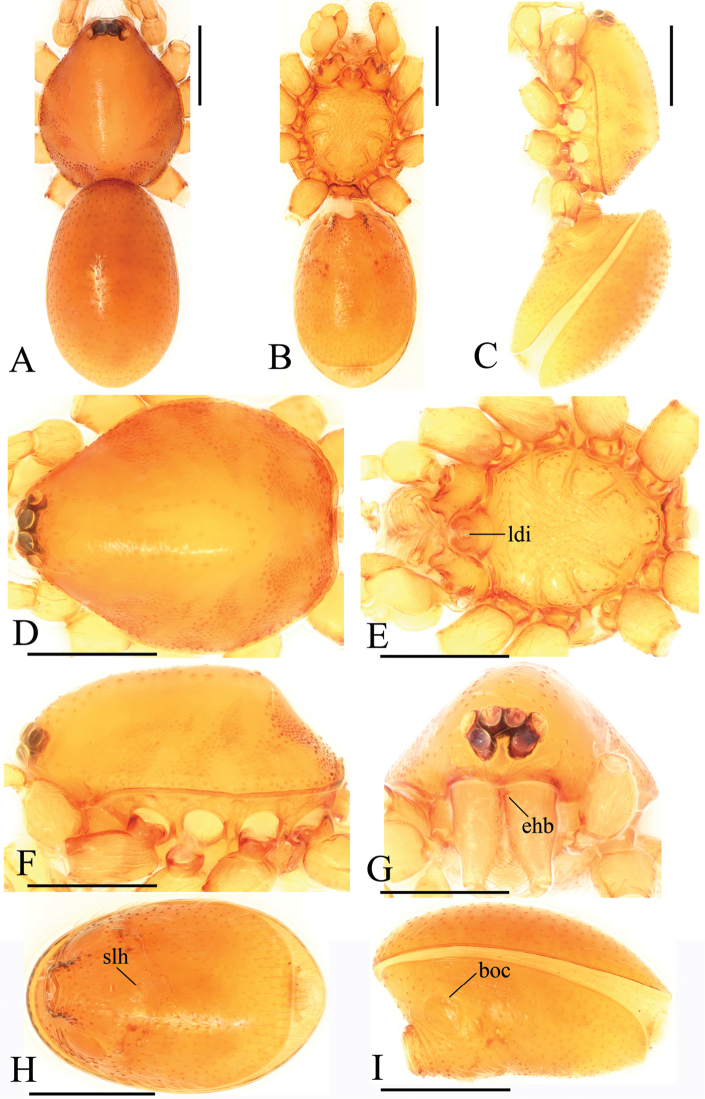
*Trilacunagongshan* sp. n., male. **A–C** habitus, dorsal, ventral and lateral views **D–G** prosoma, dorsal, ventral, lateral and anterior views **H, I** abdomen, ventral and lateral views. Abbreviations: boc = booklung covers; ehb = elevated hair base; ldi = labium deep incision; slh = small hole. Scale bars: 0.4 mm.

**Figure 11.**
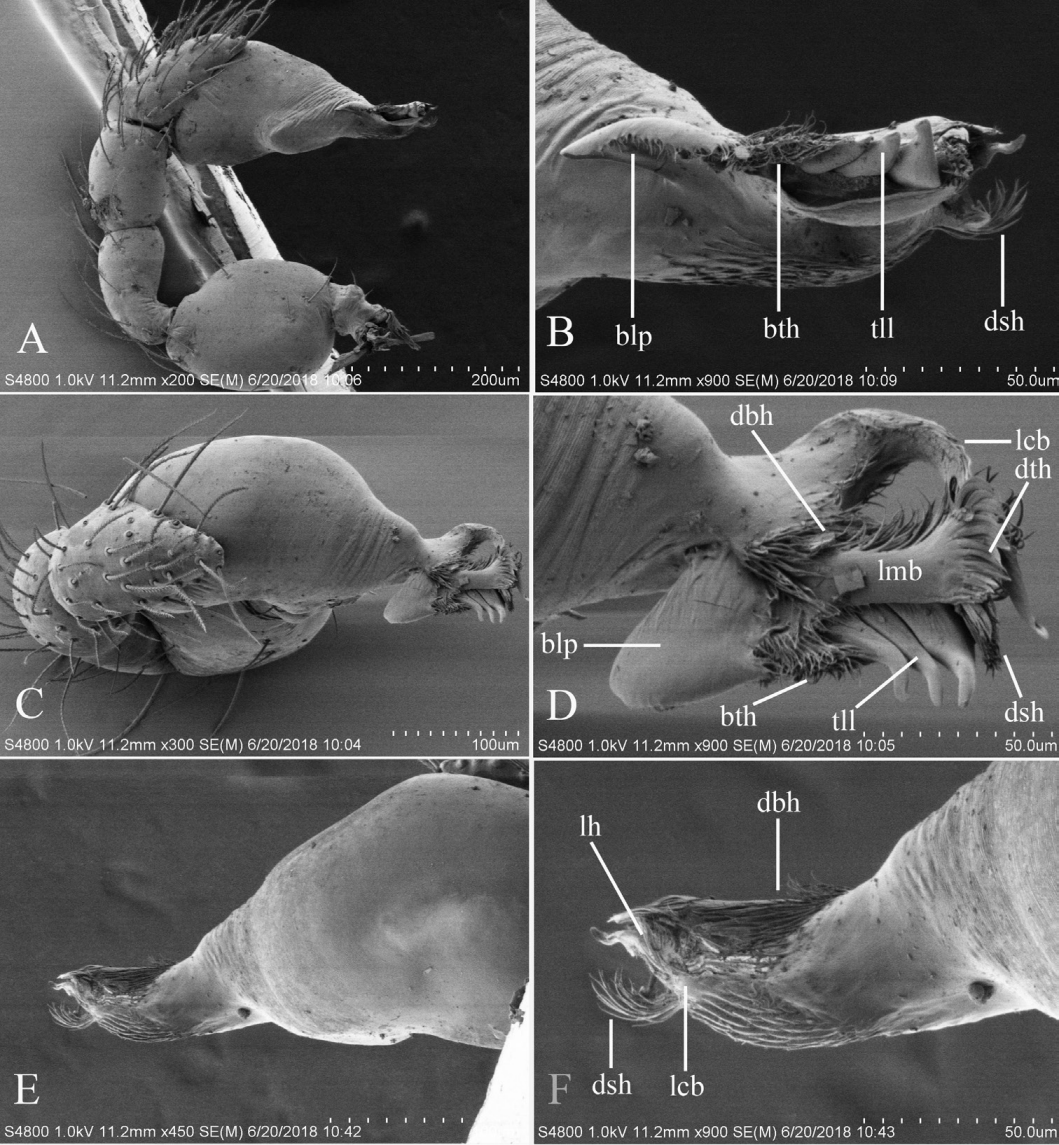
*Trilacunagongshan* sp. n., left male palp. **A** prolateral view **B, D, F** distal part of palpal bulb, prolateral, dorsal and retrolateral views **C, E** palpal bulb, dorsal and retrolateral views. Abbreviations: blp = basal leaf-shaped projection; bth = basal thin “hairs”; dbh = dorsal brush of “hairs”; dsh = distal short “hairs”; dth = distal thick “hairs”; lcb = lateral curved branch; lh = lateral “hairs”; lmb = long medial branch; tll = tooth-like lobes.

**Figure 12.**
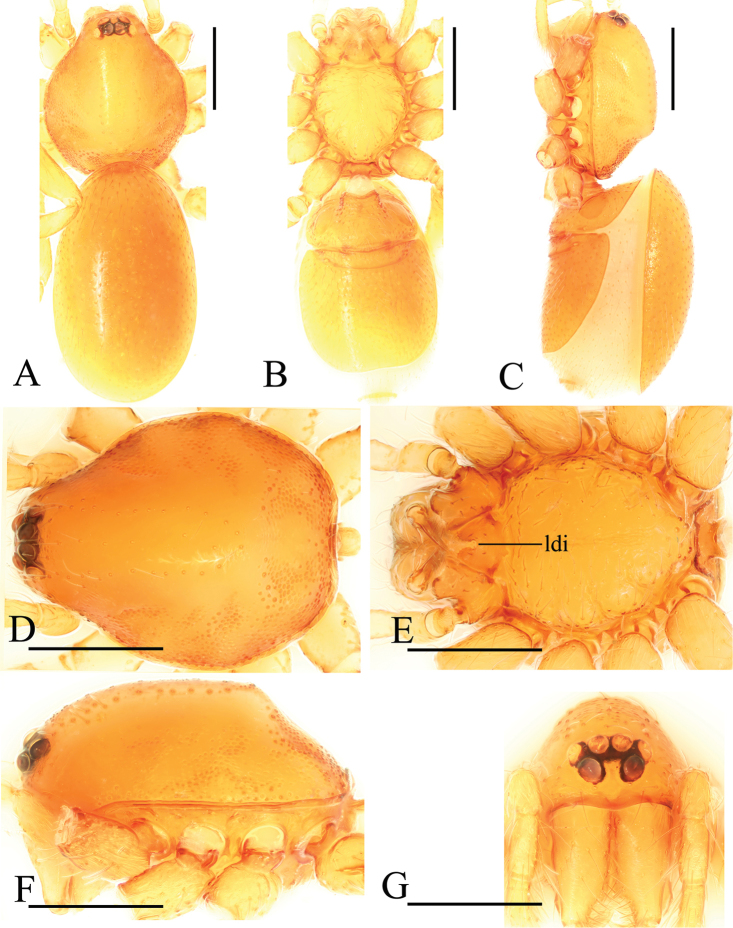
*Trilacunagongshan* sp. n., female. **A–C** habitus, dorsal, ventral and lateral views **D–G** prosoma, dorsal, ventral, lateral and anterior views. Abbreviation: ldi = labium deep incision. Scale bars: 0.4 mm.

Figs 10 , 11 , 12 , 23A, B , 24G, H , 26D

##### Type material.

**Holotype** ♂ (SYNU-257), China, Yunnan Province, Nujiang Lisu Autonomous Prefecture, Gongshan County, Bingzhongluo Town, 28°00'866"N, 98°35'953"E, 1840 m, 11.III.2011, Zongxu Li & Guchun Zhou. **Paratypes**: 6♀, 4♂ (SYNU-258), same data as holotype; 2♀, 1♂ (SYNU-259), same locality as holotype, 8.III.2011, Zongxu Li & Luyu Wang.

##### Etymology.

The specific name is a noun in apposition taken from the type locality.

##### Diagnosis.

The new specie is similar to *T.rastrum* Tong & Li, 2007, but can be distinguished by the presence of three pairs of spines on male tibiae I and II, the leaf-shaped projection (blp) and three long, tooth-like lobes (tll) on the embolus system (Fig. 11B, D), and the several longitudinal wrinkles on sternum surface of female. *Trilacunarastrum* has four pairs of ventral spines on male tibiae I and II, lacking the leaf-shaped projection, has a rake-shaped lobes on the embolus system, and has a pitted surface on sternum of female (Tong and Li 2007: fig. 7–10).

##### Description.

**Male**. Body yellow-brown, chelicerae and sternum lighter, legs yellow. Habitus as in Fig. 10A–C. Body length 1.89; carapace 0.88 long, 0.74 wide; abdomen 1.08 long, 0.68 wide. Carapace sides granulate, lateral margin rebordered (Fig. 10D). Eyes: ALE separated from edge of carapace by 1.4 diameters (Fig. 10D, G). Mouthparts (Figs 10E, G, 26D) similar to those of *T.bawan* sp. n. Sternum surface smooth, with finely setae (Fig. 10E). Abdomen as in Fig. 10I. Leg spination (all spines longer than segment width): legs I-II: tibia: v2-2-2-0, metatarsus: v2-2-0. **Genitalia**. Sperm pore situated at level of anterior spiracles; with a small hole (slh) between the posterior spiracles (Fig. 10H). Palp (Figs 11, 23A, B): orange. 0.46 long (0.15, 0.08, 0.11, 0.12). Femur 0.15 long, 0.09 wide (width/length = 0.6) (Fig. 23A, B). Cymbium without long seta. Bulb oval, stout, tapering apically. Embolus system (Fig. 11B, D, F) with a leaf-shaped projection (blp) and three long, tooth-like lobes (tll) prolaterally; with a lateral curved branch (lcb) and a long medial branch (lmb) dorsally; with numerous brush of “hairs”(dbh) surrounded medial branch and lateral “hairs” (lh) on lateral curved branch (lcb).

**Female**. As in male except as noted. Habitus as in Fig. 12A–C. Slightly larger than male. Body length 1.92; carapace 0.81 long, 0.72 wide; abdomen 1.18 long, 0.72 wide. Endites unmodified; sternum surface smooth, but medially with several longitudinal wrinkles. **Genitalia**. Ventral view (Fig. 24G): with recurved, strongly sclerotized arches (sar) anterior to the spiracles. Dorsal view (Fig. 24H): with narrow, transversally elongated sclerite (tsc); with an anterior T-shaped sclerite (as) and a posterior small globular structure (glo). Transverse bars (tba) straight, with two short, lateral apodemes (ap).

##### Distribution.

Known only from the type locality.

#### Trilacuna longling

Taxon classificationAnimaliaAraneaeOonopidae

Tong, Zhang & Li sp. n.

http://zoobank.org/68D4FDB8-9F3E-42B8-A087-977742F6ED29

**Figure 13.**
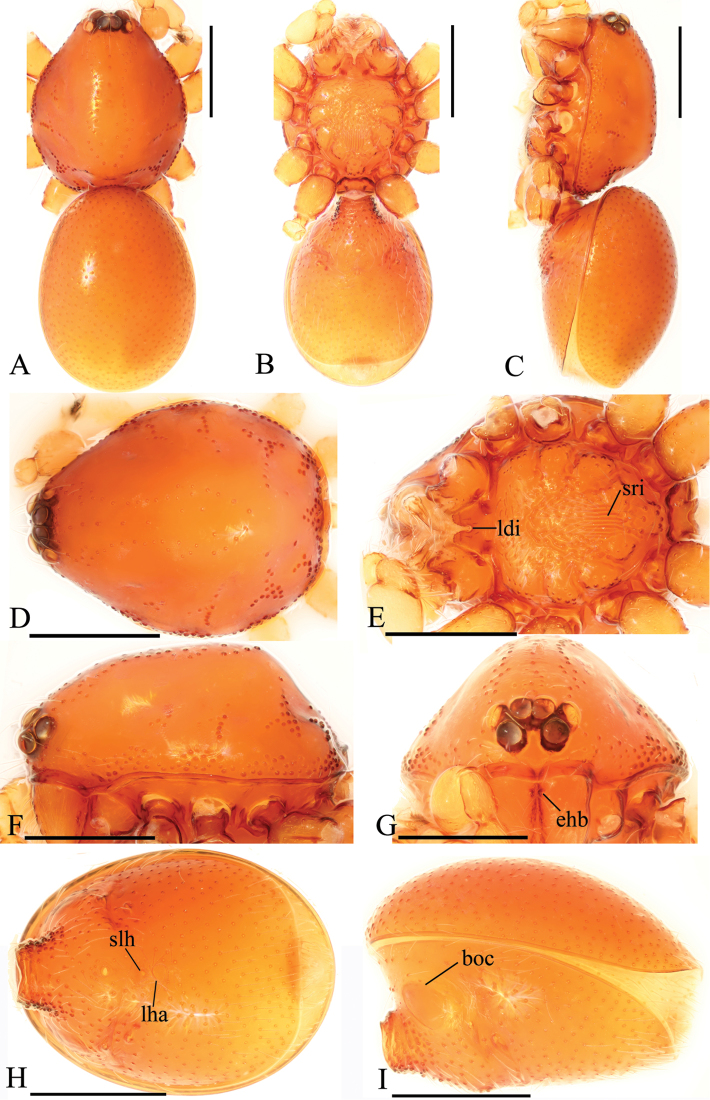
*Trilacunalongling* sp. n., male. **A–C** habitus, dorsal, ventral and lateral views **D–G** prosoma, dorsal, ventral, lateral and anterior views **H, I** abdomen, ventral and lateral views. Abbreviations: boc = booklung covers; ehb = elevated hair base; ldi = labium deep incision; lha = long hairs; slh = small hole; sri = small ridges. Scale bars: 0.4 mm.

**Figure 14.**
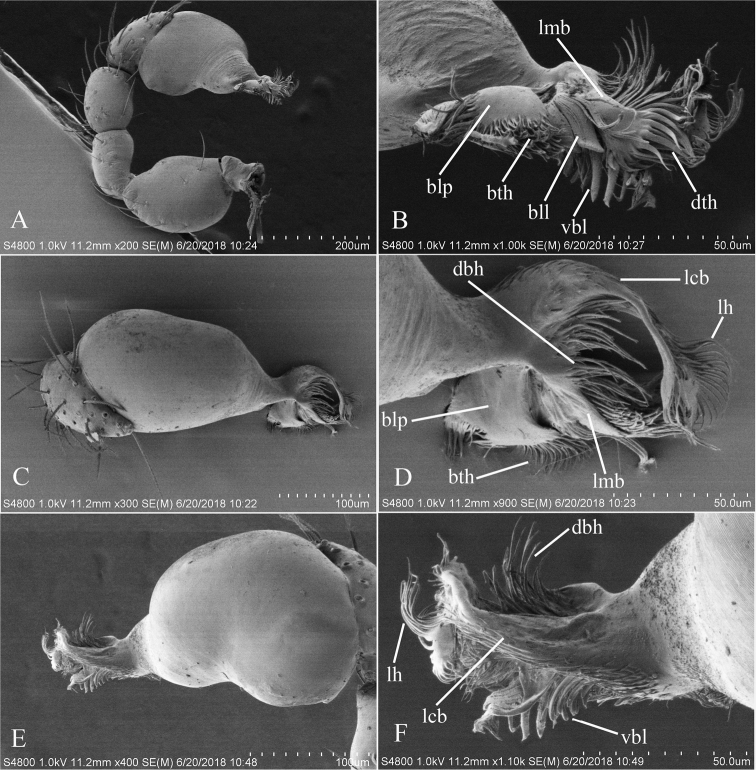
*Trilacunalongling* sp. n., left male palp. **A** prolateral view **B, D, F** distal part of palpal bulb, prolateral, dorsal and retrolateral views **C, E** palpal bulb, dorsal and retrolateral views. Abbreviations: bll = blade-like lobes; blp = basal leaf-shaped projection; bth = basal thin “hairs”; dbh = dorsal brush of “hairs”; dth = distal thick “hairs”; lcb = lateral curved branch; lh = lateral “hairs”; lmb = long medial branch; vbl = ventral broad lobes.

**Figure 15.**
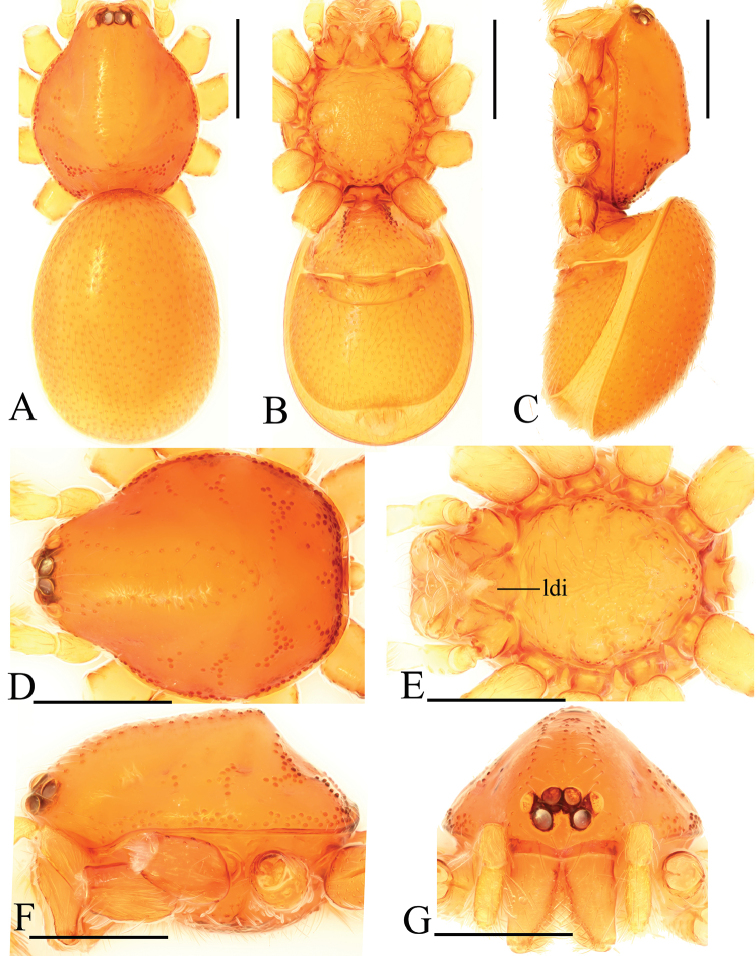
*Trilacunalongling* sp. n., female. **A–C** habitus, dorsal, ventral and lateral views **D–G** prosoma, dorsal, ventral, lateral and anterior views. Abbreviations: ldi = labium deep incision. Scale bars: 0.4 mm.

Figs 13 , 14 , 15 , 23C, D , 24I, J , 26E

##### Type material.

**Holotype** ♂ (SYNU-244), China, Yunnan Province, Baoshan City, Longling County, Xiaoheishan Natural Reserve, 17.II.2011, Zongxu Li & Luyu Wang. **Paratypes**: 2♀, 2♂ (SYNU-247), same data as holotype; 2♀ (SYNU-246), same data as holotype; 1♂ (SYNU-261), same data as holotype.

##### Etymology.

The specific name is a noun in apposition taken from the type locality.

##### Diagnosis.

The new species is similar to *T.wuhe* sp. n., but can be distinguished by the narrow, blade-like lobes (bll) on embolus system (Fig. 14B, D, F), and the straight transversal sclerite (tsc) of the endogyne (Fig. 24J). *T.wuhe* sp. n. has 4 long, finger-like lobes (fll) and a cluster of long, thick “hairs” (lth) on embolus system (Fig. 17B) and has an “angled” transversal sclerite (tsc) of the endogyne (Fig. 25A).

##### Description.

**Male**. Body yellow-brown, chelicerae and sternum lighter, legs yellow. Habitus as in Fig. 13A–C. Body length 1.69; carapace 0.81 long, 0.71 wide; abdomen 0.92 long, 0.71 wide. Carapace sides smooth, with only a few granulates, lateral margin rebordered (Fig. 13D). Eyes: ALE largest, PME smallest (Fig. 13D, G). ALE separated from edge of carapace by 1.2 diameters. Mouthparts as in Figs 13E, G, 26E. Sternum surface smooth, medial area strongly rugose, with many rows of small ridges (sri) on posterior area (Fig. 13E). Abdomen as in Fig. 13I. Leg spination (all spines longer than segment width): legs I-II: tibia: v2-2-2-2-0, metatarsus: v2-2-0. **Genitalia**. Sperm pore situated at level of anterior spiracles; with a small hole (slh) between the posterior spiracles, surrounded by some long hairs (lha) (Fig. 13H). Palp (Figs 14, 23C, D): orange. 0.46 long (0.15, 0.08, 0.11, 0.12). Femur 0.15 long, 0.09 wide (width/length = 0.6) (Fig. 23C, D). Bulb oval, stout, tapering apically. Embolus system (Fig. 14B, D, F) with a leaf-shaped prolateral projection at base (blp), projection with numerous thin, short “hairs” at the margin (bth); with two narrow, blade-like lobes (bll); with a retrolateraly curved branch (lcb) and a long medial branch (lmb), the former (lcb) with a cluster of lateral “hairs” (lh), the latter (lmb) covered by numerous dorsal “hairs” (dbh); with rows of ventral broad lobes (vbl) retrolaterally.

**Female**. As in male except as noted. Habitus as in Fig. 15A–C. Slightly larger than male. Body length 1.76; carapace 0.78 long, 0.67 wide; abdomen 1.01 long, 0.75 wide. Endites unmodified; sternum without rows of small ridges (sri) on posterior area. **Genitalia**. Ventral view (Fig. 24I): with recurved, strongly sclerotized arches (sar) anterior to the spiracles. Dorsal view (Fig. 24J): with narrow, transversally elongated sclerite (tsc); with an anterior T-shaped sclerite (as) and a posterior small globular structure (glo). Transverse bars (tba) with two short, lateral apodemes (ap).

##### Distribution.

Known only from the type locality.

#### Trilacuna wuhe

Taxon classificationAnimaliaAraneaeOonopidae

Tong, Zhang & Li sp. n.

http://zoobank.org/71684C74-2B6B-4A17-B4ED-77B51B05C535

**Figure 16.**
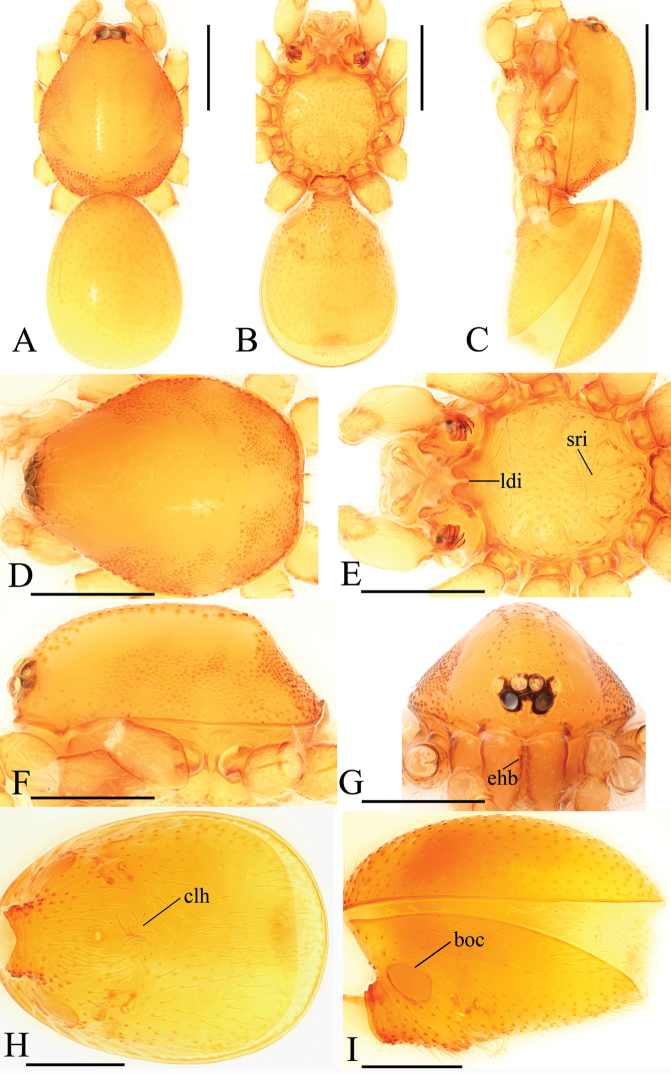
*Trilacunawuhe* sp. n., male. **A–C** habitus, dorsal, ventral and lateral views **D–G** prosoma, dorsal, ventral, lateral and anterior views **H, I** abdomen, ventral and lateral views. Abbreviations: boc = booklung covers; clh = cluster of long hairs; ehb = elevated hair base; ldi = labium deep incision; sri = small ridges. Scale bars: 0.4 mm.

**Figure 17.**
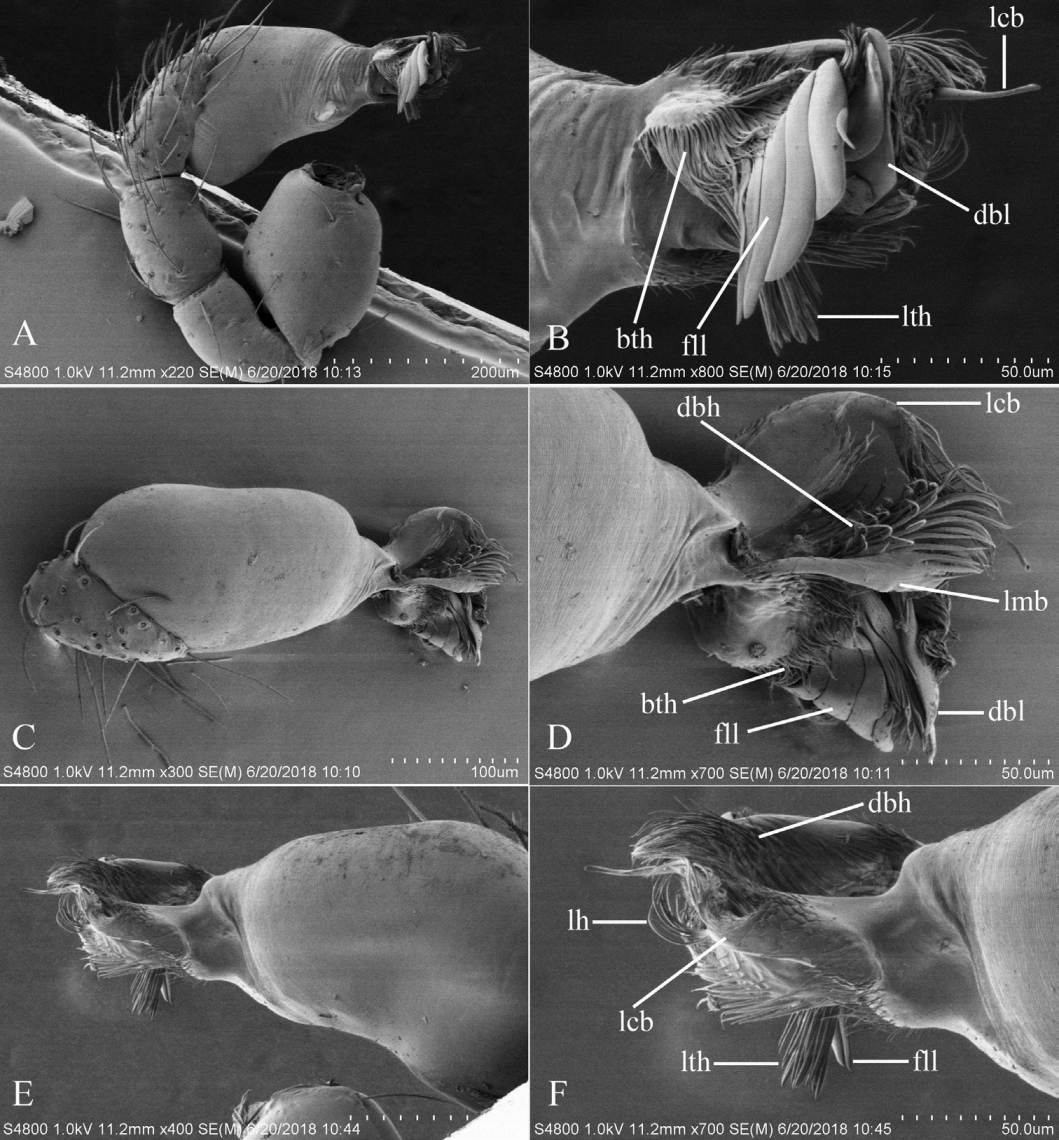
*Trilacunawuhe* sp. n., left male palp. **A** prolateral view **B, D, F** distal part of palpal bulb, prolateal, dorsal and retrolateral views **C, E** palpal bulb, dorsal and retrolateral views. Abbreviations: bth = basal thin “hairs”; dbh = dorsal brush of “hairs”; dbl = distal broad lobe; fll = finger-like lobes; lcb = lateral curved branch; lh = lateral “hairs”; lmb = long medial branch; lth = long thick “hairs”.

**Figure 18.**
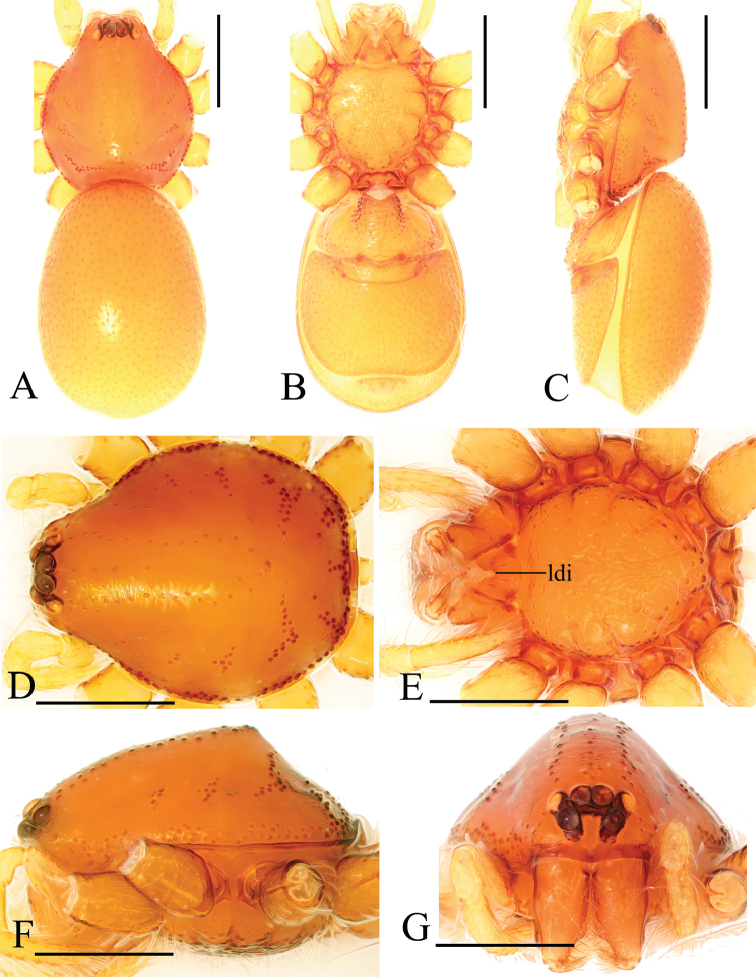
*Trilacunawuhe* sp. n., female. **A–C** habitus, dorsal, ventral and lateral views **D–G** prosoma, dorsal, ventral, lateral and anterior views. Abbreviation: ldi = labium deep incision. Scale bars: 0.4 mm.

Figs 16 , 17 , 18 , 22G–I , 25A, B , 26F

##### Type material.

**Holotype** ♂ (SYNU-255), China, Yunnan Province, Baoshan City, Tengchong County, Wuhe Town, Xiaodifang Village, 27.II.2011, Zongxu Li & Luyu Wang. **Paratypes**: 3♀, 1♂ (SYNU-256), same data as holotype.

##### Etymology.

The specific name is a noun in apposition taken from the type locality.

##### Diagnosis.

**Figure 19.**
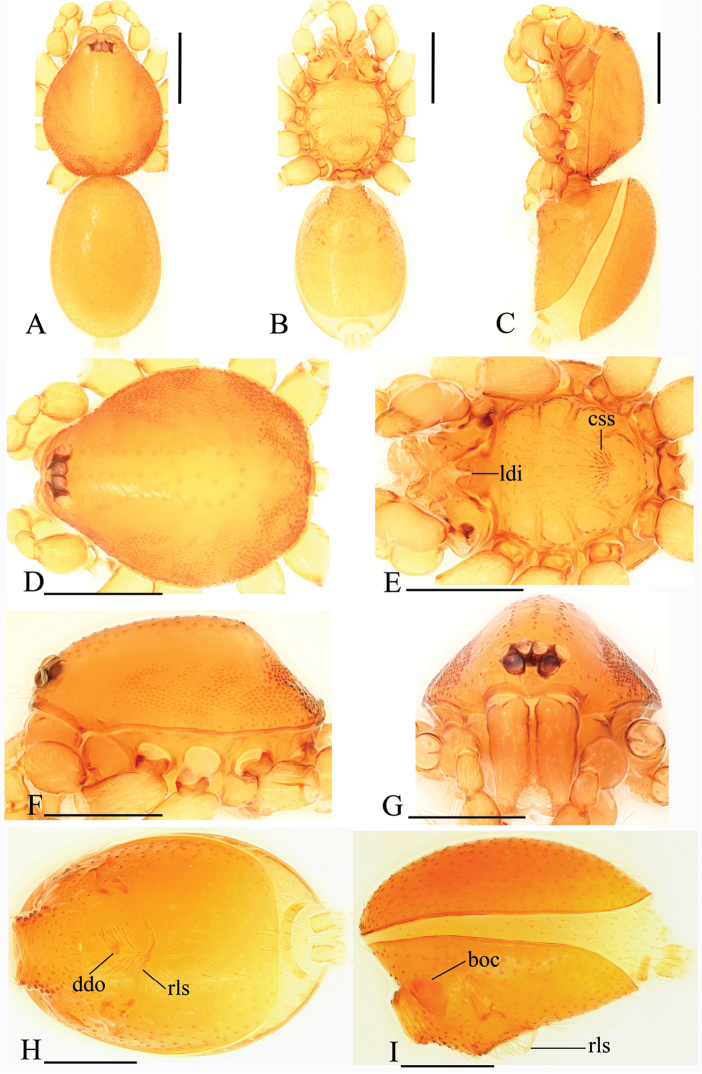
*Trilacunaxinping* sp. n., male. **A–C** habitus, dorsal, ventral and lateral views **D–G** prosoma, dorsal, ventral, lateral and anterior views **H, I** abdomen, ventral and lateral views. Abbreviations: boc = booklung covers; css = cluster of short setae; ddo = dark dot; ldi = labium deep incision; rls = rows of long setae. Scale bars: 0.4 mm.

**Figure 20.**
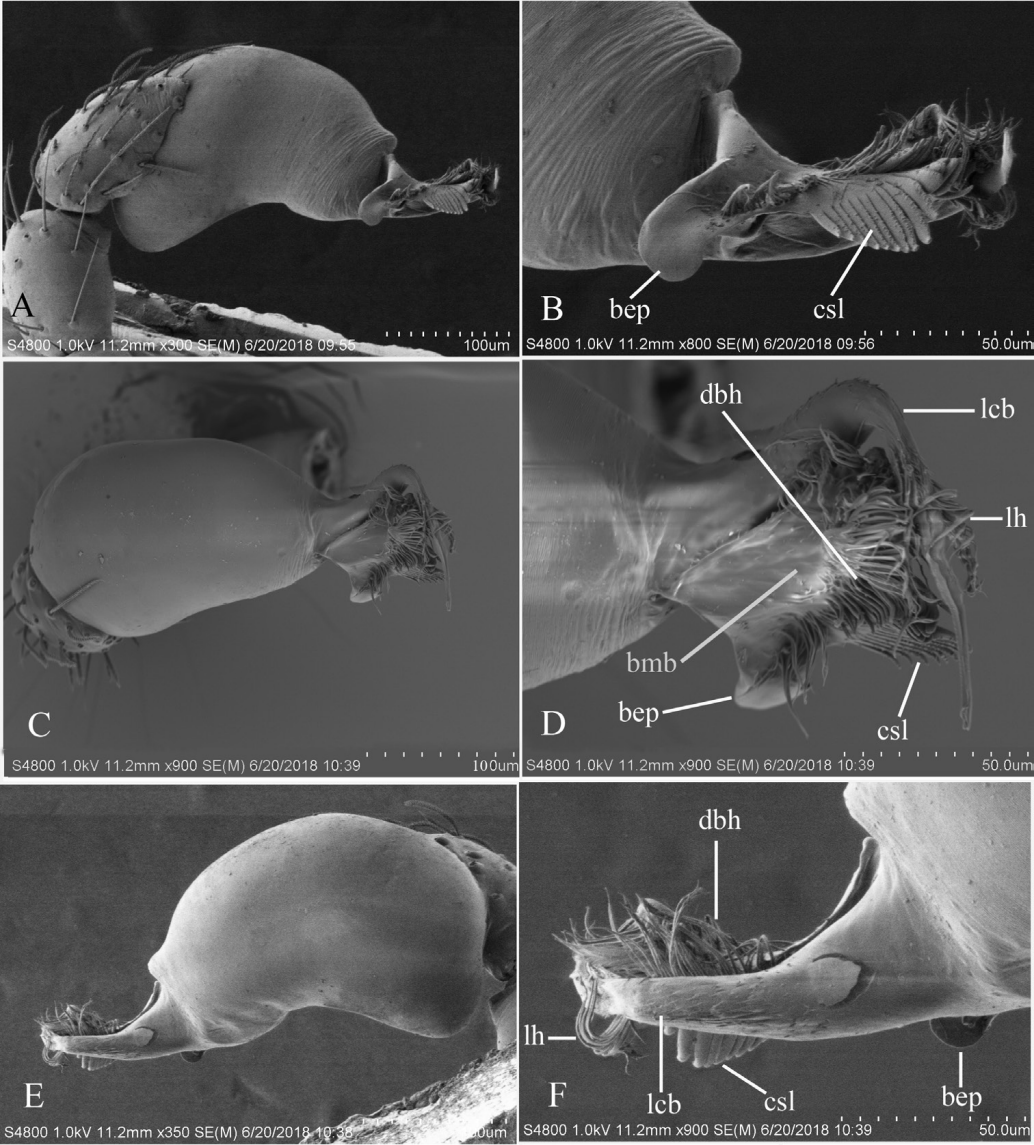
*Trilacunaxinping* sp. n., left male palp. **A** prolateral views **B, D, F** distal part of palpal bulb, prolateral, dorsal and retrolateral views **C, E** palpal bulb, dorsal and retrolateral views. Abbreviations: bep = basal ear-shaped projection; bmb = broad medial branch; csl = comb-shaped lobes; dbh = dorsal brush of “hairs”; lcb = lateral curved branch; lh = lateral “hairs”.

The new species is similar to *T.xinping* sp. n., but males can be distinguished by the numerous rows of small ridges (sri) in posterior part of sternum (Fig. 16E), four long, finger-like lobes (fll) and a cluster of long, thick “hairs” (lth) on embolus system (Fig. 17B), and the females by the “angled” transversal sclerite (tsc) of the endogyne (Fig. 25A). *Trilacunaxinping* sp. n. males have a cluster of short setae (css) in posterior part of sternum (Fig. 19E), comb-shaped lobes (csl) on embolus system (Fig. 20B), and females there is no the transversal sclerite (tsc) (Fig. 25C).

##### Description.

**Male**. Body yellow-brown, chelicerae and sternum lighter, legs yellow. Habitus as in Fig. 16A–C. Body length 1.62; carapace 0.82 long, 0.69 wide; abdomen 0.84 long, 0.64 wide. Carapace sides granulate, lateral margin rebordered (Fig. 16D). Eyes: ALE separated from edge of carapace by 1.2 diameters (Fig. 16D, G). Mouthparts as in Figs 16E, G, 26F. Sternum smooth, with many rows of small ridges (sri) on posterior area (Fig. 16E). Abdomen as in Fig. 16I. Leg spination (all spines longer than segment width): legs I-II: tibia: v2-2-2-2-0, metatarsus: v2-2-0. **Genitalia.** Sperm pore situated at level of anterior spiracles; with cluster of long hairs (clh) between the posterior spiracles (Fig. 16H). Palp (Figs 17, 22G–I): orange. 0.46 long (0.15, 0.08, 0.11, 0.12). Femur 0.15 long, 0.09 wide (width/length = 0.6) (Fig. 22G, I). Bulb oval, stout, tapering apically. Embolus system (Fig. 17B, D, F) with four long, finger-like lobes (fll), two distal broad lobes (dbl), and a cluster of long, thick “hairs” (lth) prolaterally; with a retrolateral curved branch (lcb) and a long medial branch (lmb) dorsally, all these structures surrounded by numerous hair-like structures.

**Female**. As in male except as noted. Habitus as in Fig. 18A–C. Slightly larger than male. Body length 1.81; carapace 0.79 long, 0.69 wide; abdomen 1.05 long, 0.75 wide. Endites unmodified; sternum surface slightly rugose on middle area, without rows of small ridges (sri) on posterior area. **Genitalia**. Ventral view (Fig. 25A): with recurved, strongly sclerotized arches (sar) anterior to the spiracles. Dorsal view (Fig. 25B): with narrow, nearly “angled” transversally elongated sclerite (tsc); with an anterior T-shaped sclerite (as) and a posterior small globular structure (glo). Transverse bars (tba) with two short, lateral apodemes (ap).

##### Distribution.

Known only from the type locality.

#### Trilacuna xinping

Taxon classificationAnimaliaAraneaeOonopidae

Tong, Zhang & Li sp. n.

http://zoobank.org/C5A45139-711B-4978-9026-BE121DF236B2

**Figure 21.**
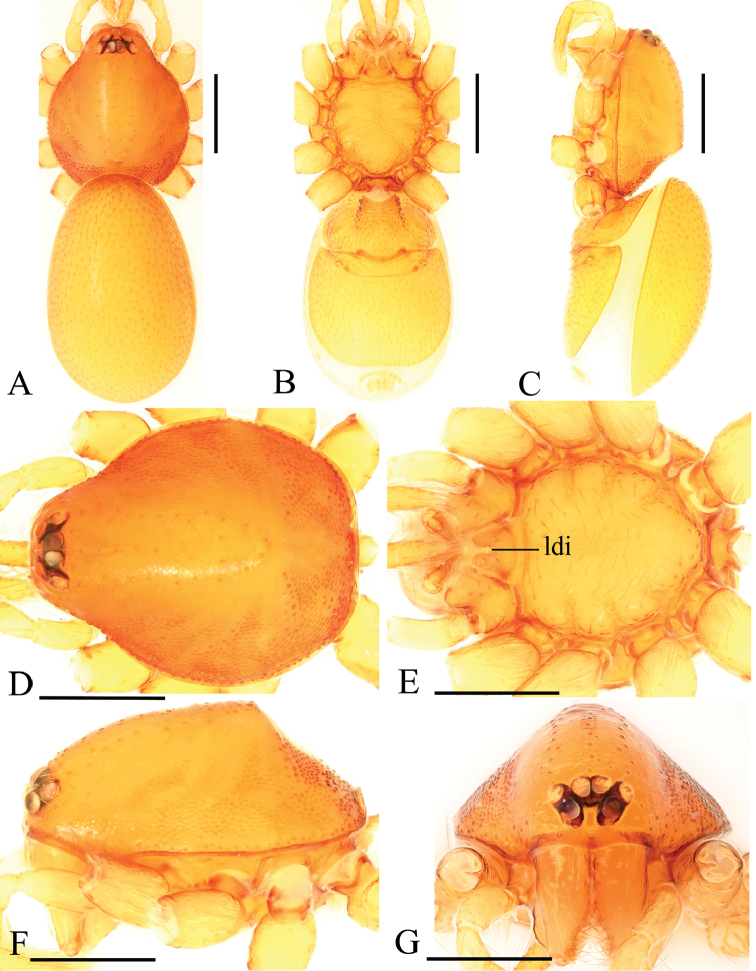
*Trilacunaxinping* sp. n., female. **A–C** habitus, dorsal, ventral and lateral views **D–G** prosoma, dorsal, ventral, lateral and anterior views. Abbreviation: ldi = labium deep incision. Scale bars: 0.4 mm.

Figs 19 , 20 , 21 , 23E–G , 25C, D , 26G

##### Type material.

**Holotype** ♂ (SYNU-248), China, Yunnan Province, Yuxi City, Xinping County, Ailaoshan Natural Reserve, on the roadside from Jinshan bealock to the Ancient Tea Horse Road, 23°56'967"N, 101°30'270"E, 2283 m, 19.V.2011, Zongxu Li & Guchun Zhou. **Paratypes**: 5♀ (SYNU-249), same data as holotype.

##### Etymology.

The specific name is a noun in apposition taken from the type locality.

##### Diagnosis.

The new species is similar to *T.rastrum* Tong & Li, 2007, but males can be distinguished by the cluster of short setae (css) on posterior part of sternum (Fig. 19E), and the kidney-shaped palpal bulb (Fig. 23E, G), and the females by the absence of the transverse sclerite (tsc) of endogyne (Fig. 25D). *T.rastrum* males are lacking cluster of short setae on posterior part of sternum, and have pear shaped palpal bulb, and the females have the transverse sclerite (Tong and Li 2007: figs 6–10).

##### Description.

**Male** (Holotype). Body yellow-brown, chelicerae and sternum lighter, legs yellow. Habitus as in Fig. 19A–C. Body length 1.74; carapace 0.81 long, 0.69 wide; abdomen 0.93 long, 0.64 wide. Carapace sides granulate. Eyes: ALE largest, PME smallest (Fig. 19D, G). ALE separated from edge of carapace by 1.1 diameters. Mouthparts as in Figs 19E, G, 26G. Sternum reticulated, with a cluster of short setae (css) posteriorly (Fig. 19E). Abdomen as in Fig. 19I. Leg spination (all spines longer than segment width): legs I-II: tibia: v2-2-2-2-0, metatarsus: v2-2-0. **Genitalia**. Sperm pore situated in front of anterior spiracles; with a small dark dot (ddo) between anterior and posterior spiracles, surrounded by rows of long setae (rls) (Fig. 19H, I). Palp (Figs 20, 23E–G): orange. Tip of cymbium with long setae, almost as long as cymbium. Bulb kidney-shaped. Embolus system (Fig. 20B, D, F), with ear-shaped projection at base (bep) and comb-shaped prolateral lobes (csl); with a lateral curved branch (lcb) and broad medial branch (bmb), the former (lcb) with a cluster of lateral “hairs” (lh), the latter (bmb) covered by numerous dorsal “hairs” (dbh).

**Female**. As in male except as noted. Habitus as in Fig. 21A–C. Slightly larger than male. Body length 1.91; carapace 0.81 long, 0.74 wide; abdomen 1.16 long, 0.76 wide. Endites unmodified; sternum without cluster of short setae on posterior area; ventral side of abdomen not elevated from lateral view. **Genitalia**. Ventral view (Fig. 25C): with recurved, strongly sclerotized arches (sar) anterior to the spiracles. Dorsal view (Fig. 25D): with an anterior T-shaped sclerite (as) and a posterior small globular structure (glo). Transverse sclerite (tsc) absent, transverse bars (tba) strongly arched, with two short, lateral apodemes (ap).

**Figure 22.**
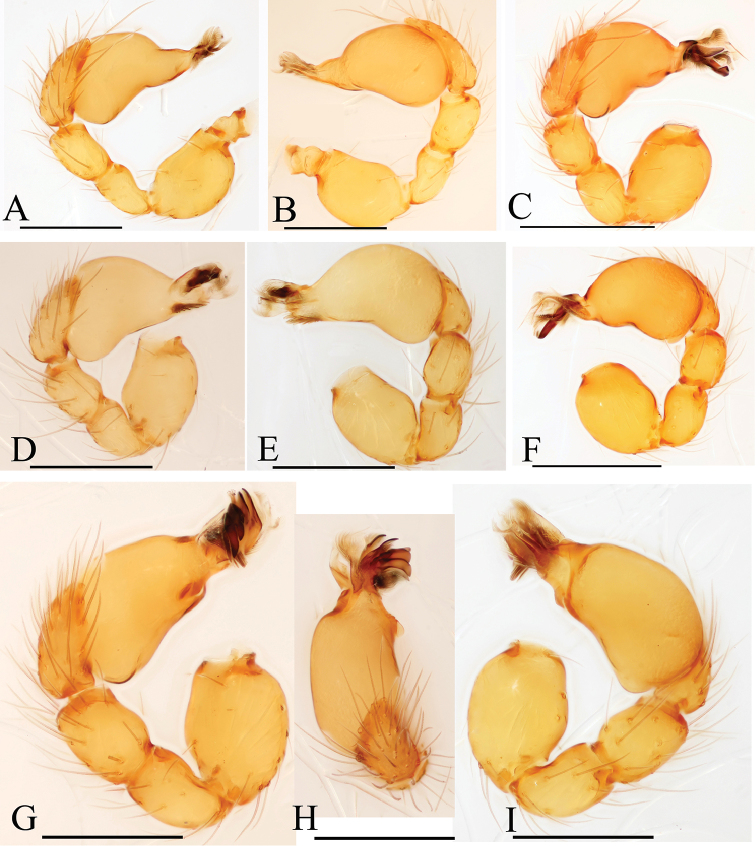
*Trilacuna* spp., male left palp. **A, B***Trilacunabawan* sp. n. **C, F***Trilacunadatang* sp. n. **D, E***Trilacunafugong* sp. n. **G–I***Trilacunawuhe* sp. n. **A, C, D, G** prolateral views **B, E, F, I** retrolateral view **H** dorsal view. Scale bars: 0.2 mm.

**Figure 23.**
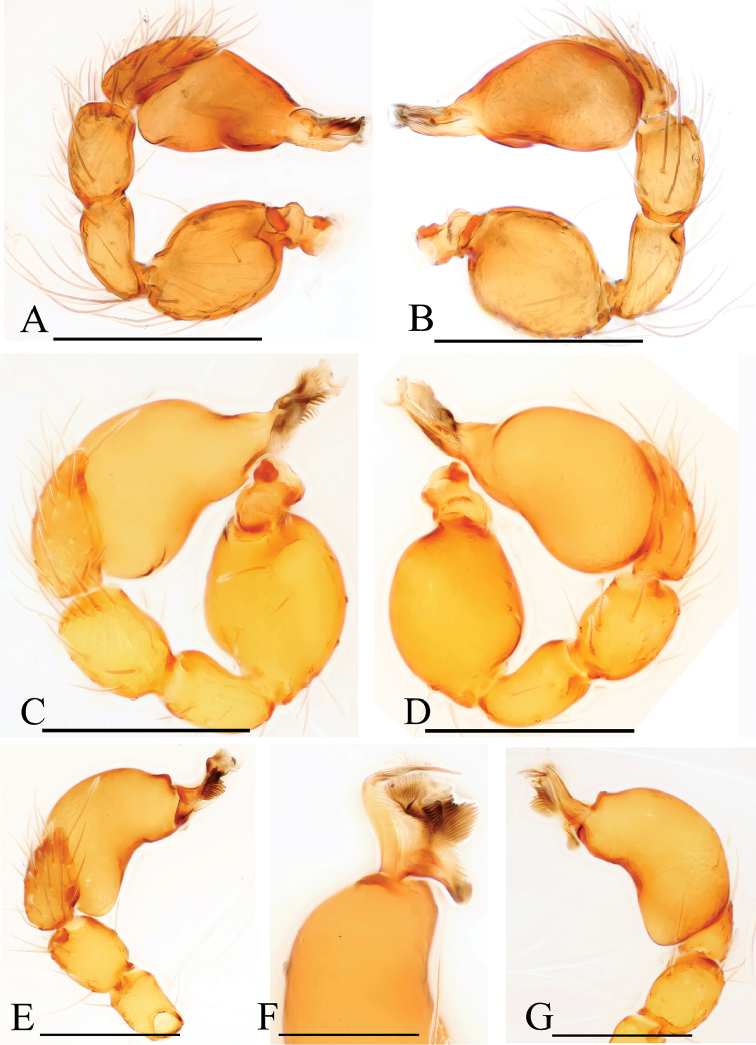
*Trilacuna* spp., male left palp. **A, B***Trilacunagongshan* sp. n. **C, D***Trilacunalongling* sp. n. **E–G***Trilacunaxinping* sp. n. **A, C, E** prolateral views **B, D, G** retrolateral view **F** dorsal view. Scale bars: 0.2 mm.

**Figure 24.**
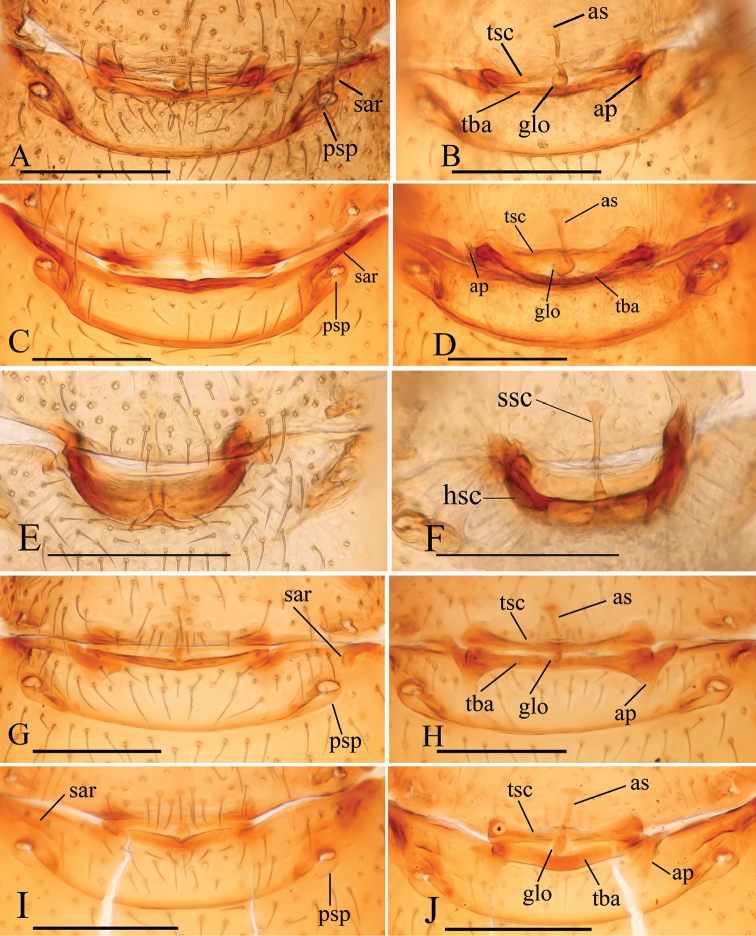
*Trilacuna* spp., female genitalia, ventral views. **A, B***Trilacunabawan* sp. n. **C, D***Trilacunadatang* sp. n. **E, F***Trilacunafugong* sp. n. **G, H***Trilacunagongshan* sp. n. **I, J***Trilacunalongling* sp. n. Abbreviations: ap = apodeme; as = anterior sclerite; glo = globular structure; hsc = horseshoe-shaped sclerite; psp = posterior spiracle; sar = sclerotized, recurved arches; ssc = stick-like sclerite; tba = transverse bars; tsc = transverse sclerite. Scale bars: 0.1 mm.

**Figure 25.**
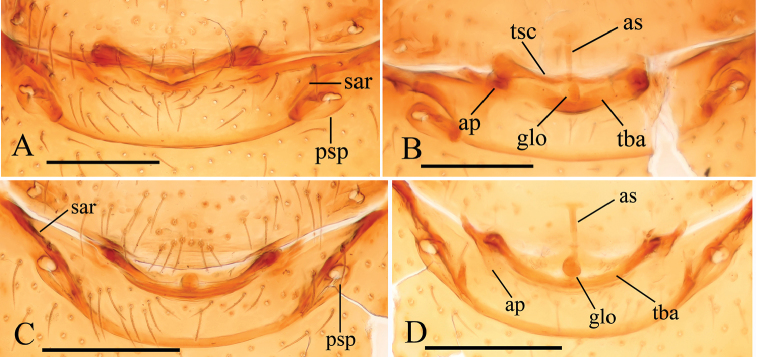
*Trilacuna* spp., female genitalia, ventral views. **A, B***Trilacunawuhe* sp. n. **C, D***Trilacunaxinping* sp. n. Abbreviations: ap = apodeme; as = anterior sclerite; glo = globular structure; psp = posterior spiracle; sar = sclerotized, recurved arches; tba = transverse bars; tsc = transverse sclerite. Scale bars: 0.1 mm.

**Figure 26.**
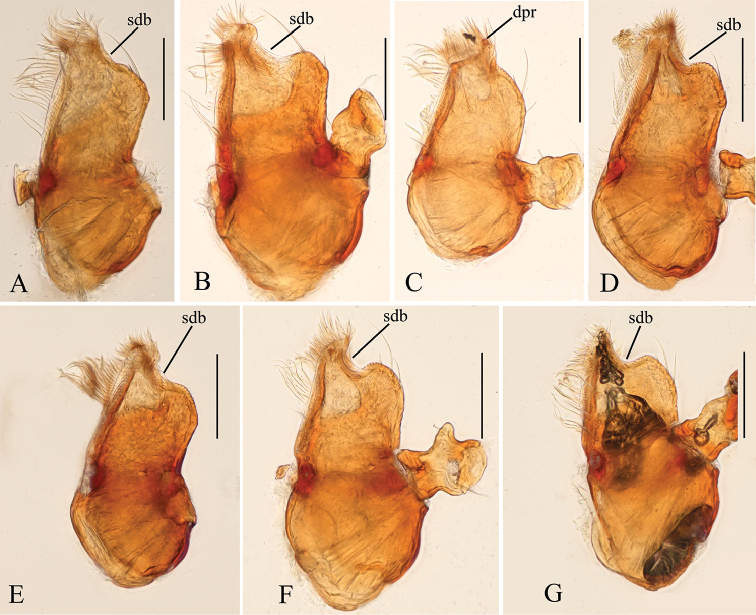
*Trilacuna* spp., male endites, ventral views. **A***Trilacunabawan* sp. n. **B***Trilacunadatang* sp. n. **C***Trilacunafugong* sp. n. **D***Trilacunagongshan* sp. n. **E***Trilacunalongling* sp. n. **F***Trilacunawuhe* sp. n. **G***Trilacunaxinping* sp. n. Abbreviations: dpr = distal projection; sdb = slightly curved distal branch. Scale bars: 0.1 mm.

##### Distribution.

Known only from the type locality.

## Supplementary Material

XML Treatment for Trilacuna

XML Treatment for Trilacuna bawan

XML Treatment for Trilacuna datang

XML Treatment for Trilacuna fugong

XML Treatment for Trilacuna gongshan

XML Treatment for Trilacuna longling

XML Treatment for Trilacuna wuhe

XML Treatment for Trilacuna xinping
